# Children With Developmental Coordination Disorder Show Altered Visuomotor Control During Stair Negotiation Associated With Heightened State Anxiety

**DOI:** 10.3389/fnhum.2020.589502

**Published:** 2020-11-27

**Authors:** Johnny V. V. Parr, Richard J. Foster, Greg Wood, Neil M. Thomas, Mark A. Hollands

**Affiliations:** ^1^Research Centre for Musculoskeletal Science and Sports Medicine, Department of Sport and Exercise Sciences, Manchester Metropolitan University, Manchester, United Kingdom; ^2^Research to Improve Stair Climbing Safety (RISCS), Faculty of Science, School of Sport and Exercise Sciences, Liverpool John Moores University, Liverpool, United Kingdom

**Keywords:** gaze, vision, kinematics, anxiety, fall-risk, fear of falling

## Abstract

Safe stair negotiation is an everyday task that children with developmental coordination disorder (DCD) are commonly thought to struggle with. Yet, there is currently a paucity of research supporting these claims. We investigated the visuomotor control strategies underpinning stair negotiation in children with (*N* = 18, age = 10.50 ± 2.04 years) and without (*N* = 16, age = 10.94 ± 2.08 years) DCD by measuring kinematics, gaze behavior and state anxiety as they ascended and descended a staircase. A questionnaire was administered to determine parents' confidence in their child's ability to safely navigate stairs and their child's fall history (within the last year). Kinematics were measured using three-dimensional motion capture (Vicon), whilst gaze was measured using mobile eye-tracking equipment (Pupil labs). The parents of DCD children reported significantly lower confidence in their child's ability to maintain balance on the stairs and significantly more stair-related falls in the previous year compared to the parents of typically developing (TD) children. During both stair ascent and stair descent, the children with DCD took longer to ascend/descend the staircase and displayed greater handrail use, reflecting a more cautious stair negotiation strategy. No differences were observed between groups in their margin of stability, but the DCD children exhibited significantly greater variability in their foot-clearances over the step edge, which may increase the risk of a fall. For stair descent only, the DCD children reported significantly higher levels of state anxiety than the TD children and looked significantly further along the staircase during the initial entry phase, suggesting an anxiety-related response that may bias gaze toward the planning of future stepping actions over the accurate execution of an ongoing step. Taken together, our findings provide the first quantifiable evidence that (a) safe stair negotiation is a significant challenge for children with DCD, and that (b) this challenge is reflected by marked differences in their visuomotor control strategies and state anxiety levels. Whilst it is currently unclear whether these differences are contributing to the frequency of stair-related falls in children with DCD, our findings pave the way for future research to answer these important questions.

## Introduction

Developmental coordination disorder (DCD) is a neurodevelopmental disorder that is estimated to affect between 1.7 and 6% of children worldwide (American Psychiatric Association, 2013). DCD is characterized by motor skill learning and performance that is far below the expected level for an individual's age and cannot be better explained by intellectual delay, visual impairment, or other neurological disorders that affect movement (Blank et al., 2019). Motor skill difficulties in DCD significantly interfere with the ability to perform activities of daily living (ADL) requiring fine and/or gross motor coordination, such as handwriting or even walking. Indeed, the walking pattern of children with DCD is often described as awkward (Gillberg and Kadesjö, 2003) and is more variable than their typically developing (TD) peers (Rosengren et al., 2009). Children with DCD also adopt a safer walking strategy during treadmill walking (Deconinck et al., 2006) and display a reduced ability to control their momentum when crossing obstacles (Deconinck et al., 2010), both of which have been attributed to a deficit to dynamic balance control. Given that children with DCD are also less accurate when tasked with precise stepping actions (Parr et al., 2020), it is unsurprising that they trip and bump into things more frequently than their TD peers (Kirby et al., 2011; Cleaton et al., 2020).

Although the difficulties children with DCD have with walking are well-documented, there is currently a paucity of research exploring how difficulties with walking translate to difficulties walking up and down stairs. This is surprising, as stair negotiation is a fundamental task that children must overcome both in and outside the home, posing a serious threat to injury in the event of a fall. Difficulties climbing stairs are commonly cited as a physical characteristic of DCD (Henderson, 1992; Missiuna et al., 2011; NHS UK, 2017) and parents of children with DCD have reported their concerns when watching their child climb stairs (Kaufman and Schilling, 2007; Missiuna et al., 2007). It has even been suggested that teachers allow children with DCD to leave class early to avoid the hazardous task of going up and down stairs when busy and cluttered with other students (Ripley, 2001). Yet, research thus far has been limited to subjective teacher reports suggesting children with DCD are less functional going up and down stairs (Wang et al., 2009) and evidence that they cannot climb as many steps as TD children in 30 s (Ferguson et al., 2014). It therefore appears that difficulties children with DCD show with balance and stability may contribute to an inherent difficulty with negotiating stairs. Consequently, there is a need to elucidate the control strategies of stair walking in children with DCD and to determine the mechanisms that may contribute to a possible increased risk of tripping and falling.

Falls most often result from a trip on a stair edge or tread surface, which likely explains why smaller step-edge clearances (i.e., distance from foot to step edge), greater clearance variability, and greater misjudgements in foot placement are linked with an increased risk of falls (Simoneau et al., 1991; Hamel et al., 2005). Stair falls are also three times more likely to occur during stair descent than stair ascent (Startzell et al., 2000), possibly reflecting the greater challenge to postural dynamic stability (Mian et al., 2007). Indeed, to recover from a loss of balance when going down the stairs, individuals must rapidly reposition their limbs whilst controlling for downwards momentum (McFadyen and Winter, 1988; Novak and Brouwer, 2011). Difficulties negotiating stairs in children with DCD may, therefore, be associated with difficulties making accurate and consistent stepping actions (Rosengren et al., 2009; Parr et al., 2020) and deficits to dynamic stability (Deconinck et al., 2006, 2010).

Accurate foot placement is also generally dependent on the appropriate use of gaze to visually extract relevant environmental features at appropriate times to optimize the planning and control of movement. For example, both older and younger adults have been shown to use gaze in a feedforward manner when navigating stairs, looking approximately three steps ahead to control their stepping behavior approximately one or two strides in advance (Zietz and Hollands, 2009; Miyasike-daSilva et al., 2011). The retrieval of feedforward visual information is particularly important at the start of a stair ascent/descent, with evidence that falls are more likely to occur when a person fails to fixate these initial, transitioning steps (Archea et al., 1979). Difficulties children with DCD display navigating stairs may, therefore, be attributable to impairments in visuomotor control and the processing of task-relevant, visual information. In tasks such as throwing and catching (Wilson et al., 2013) and sequential stepping (Warlop et al., 2020) individuals with DCD tend not to use feedforward (predictive) control to guide the planning of subsequent movements (Ferguson et al., 2015). Instead, children with DCD show a dependence on visually guided online control (Debrabant et al., 2013) despite a reduced ability to use online information to rapidly adjust and correct ongoing action (Hyde and Wilson, 2011, 2013). Consequently, children with DCD may formulate inaccurate stepping actions due to the suboptimal use of gaze to extract relevant and timely information from the environment, and the inability to predict the consequences of the ensuing movement (Wilson et al., 2013). Though our recent findings suggest that stepping inaccuracies in children with DCD may also occur despite typical looking behavior (Parr et al., 2020), it is important to determine whether the visuomotor control strategies adopted by children with DCD may be contributing to difficulties with stair negotiation.

Another factor that may contribute to difficulties negotiating stairs in children with DCD is anxiety and the fear of falling. It is well-established that fear of falling can have a concomitant impact on the visuomotor processes described above. For example, when faced with a series of obstacles, fall-related anxiety causes individuals to prioritize gaze fixations to the most proximal stepping constraints at the expense of sufficiently previewing the entire walking environment prior to negotiating it (Young et al., 2012; Ellmers and Young, 2019). Consequently, anxious individuals sometimes look away from a stepping target prematurely which results in decreased stepping accuracy and an increased risk of falling. Fall-related anxiety is also proposed to decrease attentional processing efficiency, as cognitive resources are drawn toward consciously controlling ongoing movement as opposed to carrying out concurrent processes necessary for safe locomotion, such as feedforward movement planning (Gage et al., 2003; Young and Mark Williams, 2015). To compensate for these maladaptive effects of anxiety, older adults have been shown to walk slower and increase their dynamic stability, allowing more time to plan appropriate foot placement and a heightened ability to counter forward momentum during a misstep (Novak et al., 2016; Thomas et al., under review). Evidently, anxiety is a critical factor that must be considered to fully understand the mechanisms underpinning the visuomotor control strategies of children with DCD during stair negotiation. Though we have recently shown that children with DCD do not experience heightened anxiety during over ground targeted stepping tasks (Parr et al., 2020), stair negotiation is likely to place greater demands on dynamic stability and increase the risk of significant injury in the event of a fall and may, therefore, be more likely to instill a fear response.

The aim of this study was to provide the first examination of the visuomotor control strategies that underpin stair negotiation in children with DCD and to explore the underlying influence of state anxiety. We hypothesized that the children with DCD would report heightened state anxiety and adopt a more cautious stair negotiation strategy, reflected by greater handrail use, slower walk times and more proximal gaze fixations to guide ongoing stepping commands in order to maintain stability. We also hypothesized that children with DCD would display smaller and more variable step-edge clearances compared to TD children, given their association with stair-related falls and recent evidence of decreased stepping accuracy and precision in children with DCD (Parr et al., 2020).

## Materials and Methods

### Participants

Forty-one participants aged between 8 and 15 years of age participated in the study, of which 23 were initially recruited for our DCD group. Children in the DCD group were recruited via social media and local DCD support groups and satisfied the Diagnostic and Statistical Manual of Mental Disorders (DSM-5) criteria (American Psychiatric Association, 2013). For example, parents completed the Developmental Coordination Disorder Questionnaire (DCDQ; Wilson et al., 2009) prior to testing to confirm that movement difficulties significantly interfered with their child's activities of daily living. Parents also confirmed that their child had no diagnosis of learning difficulties and did not suffer from any medical conditions known to affect sensorimotor function (e.g., cerebral palsy, hemiplegia, or muscular dystrophy). Finally, for inclusion in the DCD group, children were required to score below the 15th percentile on the test component of the Movement Assessment Battery for Children-2 (MABC-2; Henderson et al., 2007) carried out as part of the testing phase. This resulted in the data of five participants being excluded from the study (min = 25th percentile). Participants in the TD group were recruited from the family members of student and staff members at Liverpool John Moores University and were required to score above the “indication of DCD” zone of the DCDQ and above the 15th percentile of the MABC-2, resulting in one participants' data being excluded from the study. This resulted in a net total of 19 participants in the DCD group (male = 13, female = 6, age = 10.50 ± 2.04 years) and 16 participants in the TD group (male = 10, female = 6, age = 10.94 ± 2.08 years). All participants were right-foot dominant (Table 1). Parents also completed the Attentional Deficit/Hyperactivity Disorder (ADHD) Rating Scale-VI due to its high comorbidity with DCD (~50% co-occurrence; American Psychiatric Association, 2013). No child scored above the 98th percentile for inattention or hyperactivity which is recommended to be the minimum cut-off used as an indication of ADHD in research (DuPaul et al., 1998). Ethical approval was granted by the Liverpool John Moores University Ethics Committee and written informed consent was obtained from each child participant and their legal guardian prior to testing.

**Table 1:** Demographic information of the DCD and TD children included in the study.

	**DCD**	**TD**
Male (*n*)	13	10
Female (*n*)	6	6
Age (years)	10.42 ± 2.01	10.94 ± 2.08
Height (cm)	149.32 ± 10.12	146.98 ± 13.09
Weight (kg)	45.26 ± 11.41	38.95 ± 13.48
MABC-2 (%)	1.86 ± 2.75	51.31 ± 30.88

### Staircase Apparatus

**Figure 1:**
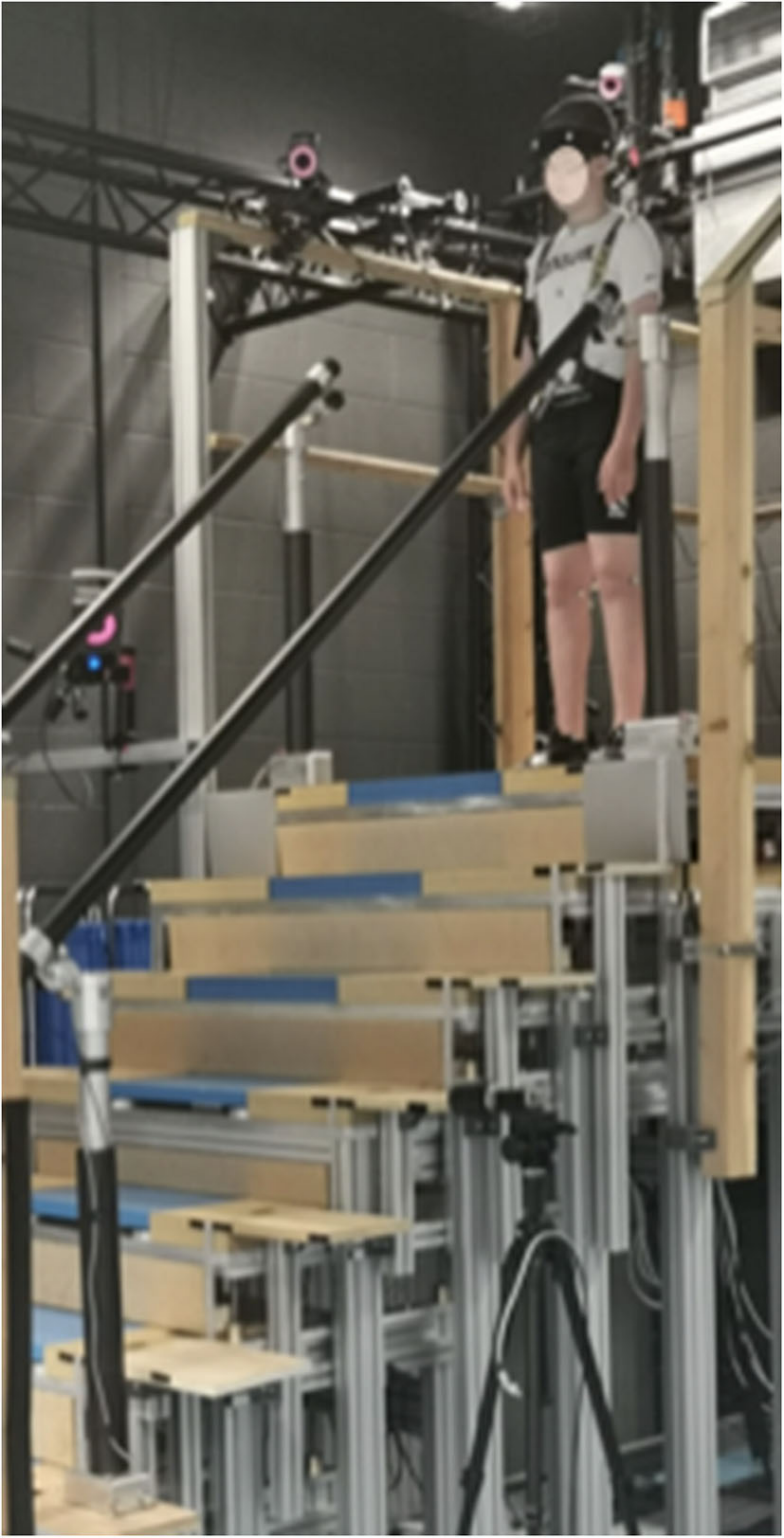
Image displaying a child participant (face blurred) preparing to descend our custom-built instrumented seven-step staircase. The child can be seen wearing a safety harness that is attached to an overhead belay safety system. Each child was allowed to freely use the handrails that can be seen either side of the staircase. The Vicon cameras can also be seen, positioned around the staircase to optimize kinematic data capture.

Participants ascended and descended a custom-made seven-step instrumented staircase with handrails on either side and a top and bottom landing long enough to enable an entry and exit phase. The riser height (20 cm) and going length (25 cm) of each step was within the current UK building regulations for commercial and private properties (Gov, 2013). Force platforms (FP; Kistler), sampling at 1,080 Hz (subsequently down sampled 120 Hz), were embedded within the bottom four steps. All participants wore a passive overhead harness whilst on the staircase that was operated by a trained belayer (Figure 1). For a detailed layout of our staircase, see Thomas et al. (2020).

### Kinematics

A 26-camera motion capture system (Vicon MX, Oxford Metrics, UK) collected whole-body kinematic data at 120 Hz, with thirty-eight reflective markers placed on the feet, lower legs, thighs, pelvis, torso and head, according to the conventional Plug-in Gait marker set. Participants wore flat footwear and tight clothing. A triangular cluster of three reflective markers (14 mm diameter) was placed on each shoe to track virtual landmarks created by a digitizing wand (C-motion, Germantown, MD, USA) at the anterior-inferior (toe-tip) and posterior inferior (heel-tip) point of each shoe. Marker trajectories were labeled, gap-filled, and exported as c3d files (Vicon Nexus 2.6, Oxford Metrics). The position of the whole-body center of mass (CoM) was estimated as the weighted sum of the various body segments using Visual3D (C-Motion, Germantown, USA). For further analysis, all kinetic and kinematic data were filtered using a phase-corrected low-pass fourth order Butterworth filter with a cut-off frequency of 6 Hz. Initial foot contacts on the staircase included contacts on the bottom landing, on each step, and on the top landing. Initial contacts on the landings were determined using local maxima of the heel referenced to the pelvis segment (Zeni et al., 2008). For the steps with no FP, local minima in the CoM vertical velocity trace defined initial contacts, and local maxima in the trailing knee flexion angle trace defined toe offs (Foster et al., 2014). For the steps with FPs, >20 N defined initial contact (Zeni et al., 2008).

### Eye Tracker

Eye movements were recorded using a Pupil Labs binocular eye-tracking mobile headset (Kassner et al., 2014) that featured two pupil cameras that recorded pupil movements at 60 Hz, and a scene camera to record the world view at 30 Hz. Prior to task performance, participants completed a 5-point screen marker calibration that was re-run when the calibration had been visibly lost. If the child failed calibration after multiple attempts, or persistently lost calibration due to excessive movement of the headset, the task was run without the eye tracker and their gaze data excluded. Participants' gaze data were also only included in the analyses of each condition if they presented at least two usable trials. Consequently, eye-movement data from two TD participants (male = 1, female = 1, age = 9.50 ± 0.71, MABC-2 = 25.00 ± 00.00) were excluded for the descent condition, and eye-movement data from four DCD participants (male = 2, female = 2, age = 9.50 ± 1.29, MABC-2 = 3.37 ± 3.82) were excluded from both the ascent and descent conditions. From the participants included in gaze analyses, an average of 7 ± 12% of trials were excluded for the stair ascent, and an average of 12 ± 18% of trials were excluded for the stair descent. Eye tracking footage was also used to determine whether a particular trial did or did not involve some use of the handrails.

### Parental Confidence Questionnaire

All parents completed a 9-question survey designed to examine (a) the confidence they have in their child's ability to safely ascend and descend stairs, and (b) how frequently their child experiences stair-related falls in everyday life. The first eight questions consisted of a Likert scale ranging from zero (not confident at all) to one-hundred (absolutely confident) and probed how confident each parent was that their child could (Q1/2) go up/down the stairs without losing balance (typical stair negotiation), (Q3/4) go up/down the stairs rapidly without losing balance (rapid stair negotiation), (Q5/6) go up/down the stairs without a handrail and not lose balance, and (Q7/8) recover from a loss of balance going up/down the stairs to prevent a fall. The final question (Q9) asked each parent to provide an estimation of the total number of stair-related falls (going up or down) their child had experienced in the year prior to testing.

### State Anxiety Questionnaire

State anxiety levels were measured using a child friendly “fear thermometer” (www.anxietycanada.com) which encompasses a 10-point “smiley-face” Likert scale ranging from 1 (low levels of anxiety) to 10 (high levels of anxiety). These simple scales have previously been validated against larger and more complex state anxiety inventories (Houtman and Bakker, 1989).

### Protocol

Data collection took place in a single session lasting ~2-h. Once prepared for kinematic and gaze data collection, baseline levels of state anxiety were collected. After a demonstration by the experimenter, participants were then instructed to ascend and descend the stairs at a steady self-selected pace and to avoid running or jogging up/down the stairs. All analyzed trials were performed in a step-over-step fashion, placing only a single foot on each step (as per the demonstration). A total of three trials were removed from analysis due participants exhibiting some step-by-step walking, placing each foot on a single step. Participants performed 5 trials in each direction (ascent and descent), starting with ascent. At the beginning of each trial, participants stood facing the staircase on either the lower (ascent) or upper (descent) landing with their feet side-by-side whilst maintaining their gaze on a red light-emitting-diode (LED) positioned to the left of each starting position (Figure 1). This ensured standardization of visuomotor planning across participants prior to each trial. The red LED turned off at the onset of kinematic data collection (initiated by Vicon) and acted as a “go” signal for participants to begin each trial, at which point they could look where they wanted. To ensure ecological validity, participants were free to use the handrails throughout. Once participants had ascended/descended the staircase, they were instructed to continue walking along the landing before coming to a stationary position. Immediately prior to the first ascent and first descent trial, state anxiety was again measured to determine task-specific fluctuations in anxiety.

### Data Analysis

#### Kinematic Variables

Stair ascent and descent durations were calculated as the interval (in seconds) between the foot contacts occurring on the first and seventh step steps. The interval between foot contacts occurring on subsequent steps (“step duration”) was also measured to determine how particular sections on the staircase may contribute to overall stair ascent/descent durations. The variability in both ascent/descent durations and step duration were calculated as the standard deviation of values across trials. To characterize whether children with DCD showed lower postural stability and reduced stepping control during walking compared with TD children we measured margin of stability, foot-step-edge clearances and foot-step-edge clearance variability. These measures were identified due to their association with fall-risk on stairs. Margin of stability was expressed as the distance between the extrapolated CoM (xCoM) and the forward boundary of the base of support (in this instance, the toe-marker). When the toe-marker was overhanging the confines of the step-edge, the forward boundary was instead defined as the step-edge. Smaller (or more negative) margins of stability are considered to reflect a less dynamically stable pattern of stair negotiation (Bosse et al., 2012; Novak et al., 2016). Margin of stability has been shown to increase in older adults under conditions of poor lighting (Thomas et al., 2020) and ambiguous carpet patterns (Thomas et al., under review) when descending stairs, and can therefore detect safety-related adaptations to stair navigation strategy. *xCoM* was defined as

$$xCoM=pCoM+vCoM/\sqrt{(gl^{-1}})$$

where *pCoM* is the AP position of the CoM, *vCoM* is the instantaneous AP velocity of the CoM, *g* is acceleration due to gravity, and *l* is the absolute distance between the CoM and the ankle joint center. Margin of stability was calculated at initial contact on each of the seven steps (Debelle et al., 2020). For ascent, foot-step-edge clearance was defined as the minimum vertical distance between toe markers on the lead limb and the step edges (toe clearance). For descent, foot-step-edge clearance was defined as the minimum horizontal distance between heel markers on the lead limb and the step edges (heel clearance). Foot-step-edge clearance variability was measured as the standard deviation of step-edge clearances across trials on each step. Increased clearance variability is proposed to increase the risk of catching the foot on a step edge and thus cause a fall/loss of balance.

#### Gaze Variables

Frame-by-frame analysis of eye-tracking video footage was performed to identify gaze location from trial onset (identified by the LED “go” signal) until trial offset (foot contact on the seventh step in sequence). Gaze fixations were defined as a gaze stabilization on a location in the environment for three frames or longer (corresponding to ~100 ms). Fixations were classified as being spatially located on one of the following areas of interest: (1) *bottom of the staircase (Bottom)*: one tread-length before the stairs and step 1; (2) *lower mid-steps (M1)*: steps 2 and 3; (3) *upper mid-steps (M2)*: steps 4 and 5; (4) *top of the staircase (Top)*: steps 6 and 7; (5) *far landing*: incorporating fixations on the far landing and on the back wall (relative to either the stair ascent or descent), and (6) *handrails*. To understand how gaze was allocated across each individual phase of movement (i.e., “gaze in action”), we expressed total time fixating each AOI during each phase of movement as a percentage of total phase duration (Miyasike-daSilva et al., 2011). We also measured the average number of steps the participants looked ahead during each phase of movement. In line with previous research (Zietz and Hollands, 2009), we considered participants to be looking one step ahead when they were fixating the next step in sequence. For example, a person who has just made foot contact on step 3 would be looking one step ahead if fixating step 4 but looking two steps ahead if fixating step 5. Other gaze variables included average fixation duration and fixation rate (number of fixations divided by the total stair ascent/descent duration, expressed as fixations per second).

### Statistical Analyses

All analyses were performed and are presented separately for the stair ascent and stair descent. Independent samples *t-*tests were run to compare stair ascent/descent duration, fixation duration and fixation rate between groups. Effect sizes were expressed using Cohen's *d*, with common indicative thresholds reported as small (0.2), medium (0.5), and large (0.8). Kinematic variables characterizing risky stair behavior (step duration; foot clearances; foot clearance variability; margin of stability) were analyzed using a two-way mixed design repeated measures ANOVA, with between-subject effects of Group (x2 DCD; TD) and within-subject effects of Phase (x4; Bottom, M1, M2, Top). A two-way repeated measures ANOVA was also used to examine how many steps participants looked ahead throughout each trial, with between-subject effects of Group and within-subject effects of Phase (x4; Bottom; M1; M2; Top). Significant effects were probed by polynomial trend analyses, and *post-hoc* analyses were performed using pairwise comparisons with Sidak-corrections to account for the multiple comparisons problem (Blakesley et al., 2009). ANOVA effect sizes were reported using partial eta squared (η_p_^2^), common indicative thresholds for which are small (0.01), medium (0.06) and large (0.14; (Field, 2013). The results of univariate tests are reported, with Huyn-Feldt correction procedure applied for analyses that violated sphericity of variance. Where data were not normally distributed, within participant effects were analyzed using Friedman's ANOVA, and Bonferroni corrected Wilcoxon-signed rank tests for *post-hoc* analyses. Between-subject effects were analyzed using Mann-Whitney *U-*tests. Non-parametric effect sizes were reported as *r* = *Z*/$\sqrt{n}$, for which common thresholds are small (0.1), medium (0.3) and large (0.5; Rosenthal, 1986). All statistical analyses were performed using IBM SPSS statistics (version 26) with an alpha level of ≤ 0.05.

## Results

### Parent Confidence Questionnaire

Results from separate Mann-Whitney *U-*tests showed the parents of children with DCD reported significantly lower confidence in their child's ability to use the stairs compared to the parents of TD children for all included scenarios (*ps* < 0.001). Compared to the parents of TD children (*Median* = 0.0, *Mean (M)* = 2.1 ± 5.2), the parents of DCD children (*Median* = 3.5, *M* = 12.3 ± 31.9) also reported significantly more stair-related falls in the last year (*U* = 69.50, *p* = 0.034, *Z* = −2.123; Table 2).

**Table 2:** Mean (SD) levels of confidence reported by the parents of children with and without DCD across each stair-specific walking scenario during both stair ascent and stair descent.

	**Confidence (%)**			
	**Ascent**		**Descent**	
**Stair walking scenario**	**DCD**	**TD**	**DCD**	**TD**
Typical stair negotiation (Q1/2)	76.5 (21.32)*	96.80 (4.69)	67.68 (28.97)*	96.00 (5.63)
Rapid stair negotiation (Q3/4)	62.56 (27.59)*	92.66 (7.31)	51.5 (28.83)*	91.73 (8.28)
Without handrails (Q5/6)	47.94 (30.83)*	95.13 (5.84)	40.38 (29.42)*	94.07 (6.85)
Recover from loss of balance (Q7/8)	53.44 (30.33)*	90.2 (11.54)	44.38 (27.53)*	88.2 (14.64)

*Asterisks indicate significant between group differences (p < 0.001)*.

### Stair Ascent

There were no significant Group x Phase interactions for any ascent outcome measures.

#### Anxiety

Results showed no significant difference between groups for levels of state anxiety prior to the first stair ascent (*U* = 128.00, *z* = −0.874, *p* = 0.382, *r* = −0.148).

#### Handrail Use

Children with DCD displayed greater frequency of handrail use than the TD children (*U* = 81.00, *p* = 0.003, *r* = 0.495). Two TD children used the handrails during the stair ascent, both using the handrail for all 5 trials. In comparison, 14 DCD children used the handrails, 10 using the handrails for all 5 trials.

#### Stair Ascent Duration

**Figure 2:**
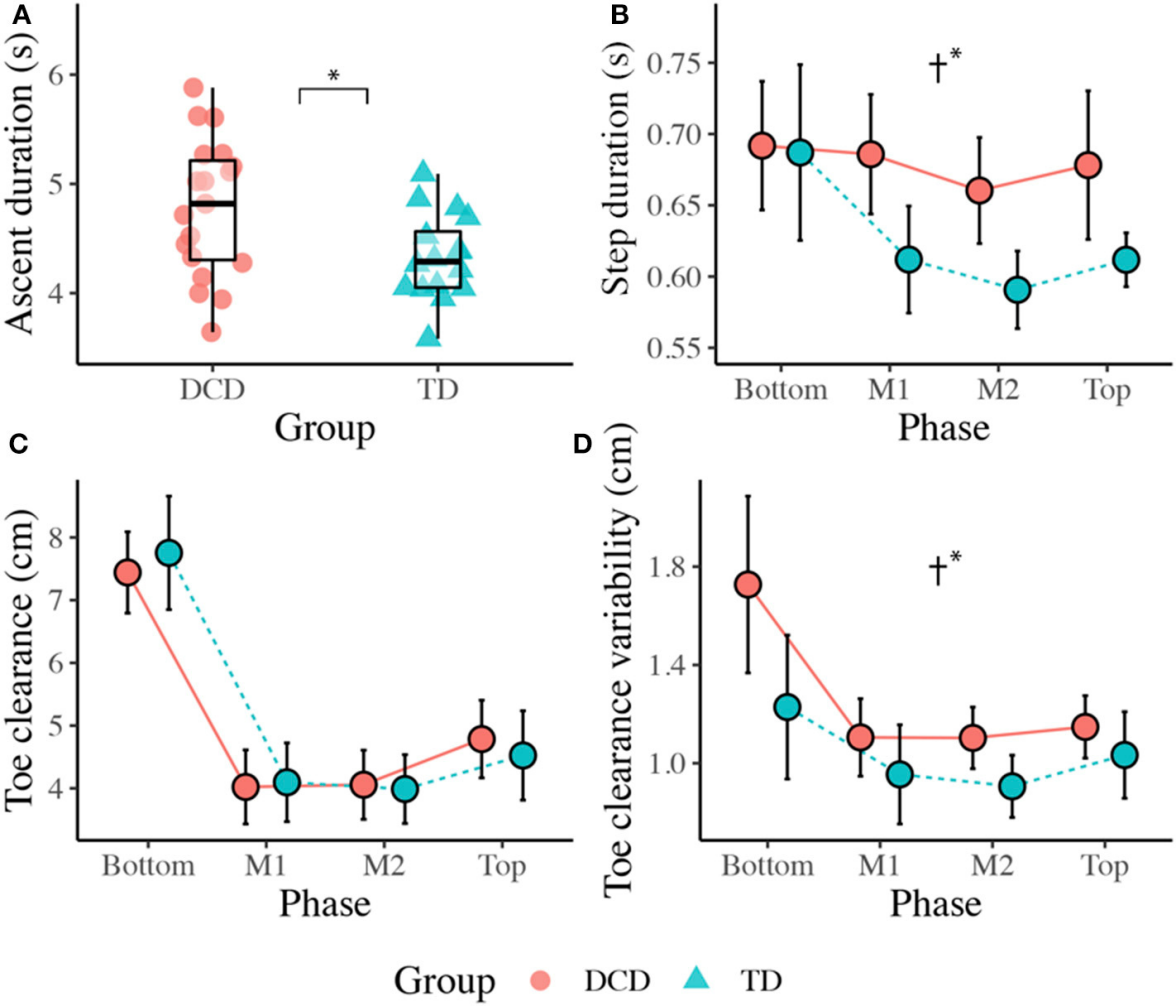
Boxplots displaying the median, quartiles, and each individual's mean time taken to ascend the staircase **(A)**, and line plots displaying the mean (±95% CI) time taken to complete each step **(B)**, the mean (±95% CI) vertical toe clearance **(C)**, and the mean vertical toe clearance variability **(D)** for both DCD and TD children across each phase of the staircase. † Significant main effect of Group (*p < 0.05).

Children with DCD (*M* = 4.78 s) took significantly longer than the TD children (*M* = 4.32 s) to ascend the staircase, *t*(33) = 2.596, *p* = 0.014, *d* = 0.861. However, ascent duration variability did not significantly differ between the DCD (*M* = 0.46 s) and TD (*M* = 0.36 s) groups, *U* = 109.00, *z* = −1.424, *p* = 0.154, *r* = −0.248 (Figure 2).

#### Step Duration

There was a significant main effect of Group, *F*_(1, 33)_ = 4.892, *p* = 0.034, η_p_^2^ = 0.129, with greater step durations observed in the DCD children (*M* = 679 ms) compared to the TD children (*M* = 625 ms). There was also a significant main effect of Phase, *F*_(1.559, 51.447)_ = 5.879, *p* = 0.009, η_p_^2^ = 0.151, with *post-hoc* analyses revealing significantly longer step durations at the Bottom of the staircase (*M* = 689 ms) compared to the M2 phase (*M* = 626 ms, *p* = 0.007). There was no Phase x Group interaction, *F*_(3, 99)_ = 2.187, *p* = 0.094, η_p_^2^ = 0.062. For step duration variability, there was no main effect of Group, *F*_(1, 33)_ = 1.399, *p* = 0.245, η_p_^2^ = 0.041, no main effect of Phase, *F*_(2.132, 70.343)_ = 1.887, *p* = 0.157, η_p_^2^ = 0.054, and no Phase x Group interaction, *F*_(3, 99)_ = 0.802, *p* = 0.496, η_p_^2^ = 0.024 (Figure 2).

### Kinematics

#### Vertical Toe Clearance

There was no effect of Group, *F*_(1, 33)_ = 0.001, *p* = 0.971, η_p_^2^ = 0.000, but there was a significant main effect of Phase, *F*_(1.656, 54.646)_ = 118.584, *p* < 0.001, η_p_^2^ = 0.782. Comparisons revealed greater vertical toe clearance at the Bottom (*M* = 7.6 cm, *ps* < 0.001) and Top (M = 4.7 cm, *ps* < 0.017) of the staircase compared to M1 (*M* = 4.1 cm) and M2 (*M* = 4.0 cm; Figure 2).

#### Vertical Toe Clearance Variability

There was a significant main effect of Group, *F*_(1, 33)_ = 6.601, *p* = 0.015, η_p_^2^ = 0.167, with greater variability observed in the DCD group (*M* = 1.3 cm) compared to the TD group (*M* = 1.0 cm). There was also a significant main effect of Phase, *F*_(1.934, 63.828)_ = 9.673, *p* < 0.001, η_p_^2^ = 0.227, with *post-hoc* comparisons revealing greatest variability at the Bottom of the staircase (*M* = 1.5 cm) compared to all other phases (*ps* < 0.015; Figure 2).

#### MoS Anteroposterior

There was no main effect of Group, *F*_(1, 33)_ = 1.092, *p* = 0.304, η_p_^2^ = 0.032. There was a significant main effect of Phase, *F*_(1.382, 45.599)_ = 4.970, *p* = 0.021, η_p_^2^ = 0.131, but comparisons did not reveal any significant differences across phase pairs.

### Gaze Behavior

#### Fixation Rate/Duration

Independent-samples *t-*tests showed no significant difference between the TD (*M* = 2.21) and DCD (*M* = 2.08) groups for fixation rate, *t*(31) = 1.076, *p* = 0.290, *d* = 0.37, and no difference between the TD (*M* = 309.03 ms) and DCD (*M* = 319.12 ms) groups for mean fixation duration, *t*(31) = −0.818, *p* = 0.420, *d* = 0.28.

#### Steps Looked Ahead

There was no main effect of Group, *F*_(1, 31)_ = 0.025, *p* = 0.874, η_p_^2^ = 0.001, but a significant main effect of Phase, *F*_(2.137, 66.233)_ = 41.635, *p* < 0.001, η_p_^2^ = 0.573, that was best described by a quadratic linear trend, *F*_(1, 31)_ = 102.692, *p* < 0.001, η_p_^2^ = 0.768, indicating that participants looked fewer steps ahead during the initial Bottom phase (*M* = 2.20), more steps ahead during the M1 (*M* = 3.12), and M2 (*M* = 3.204) phases, and then fewer steps ahead during the final Top phase (*M* = 2.05).

#### Gaze in Action

**Figure 3:**
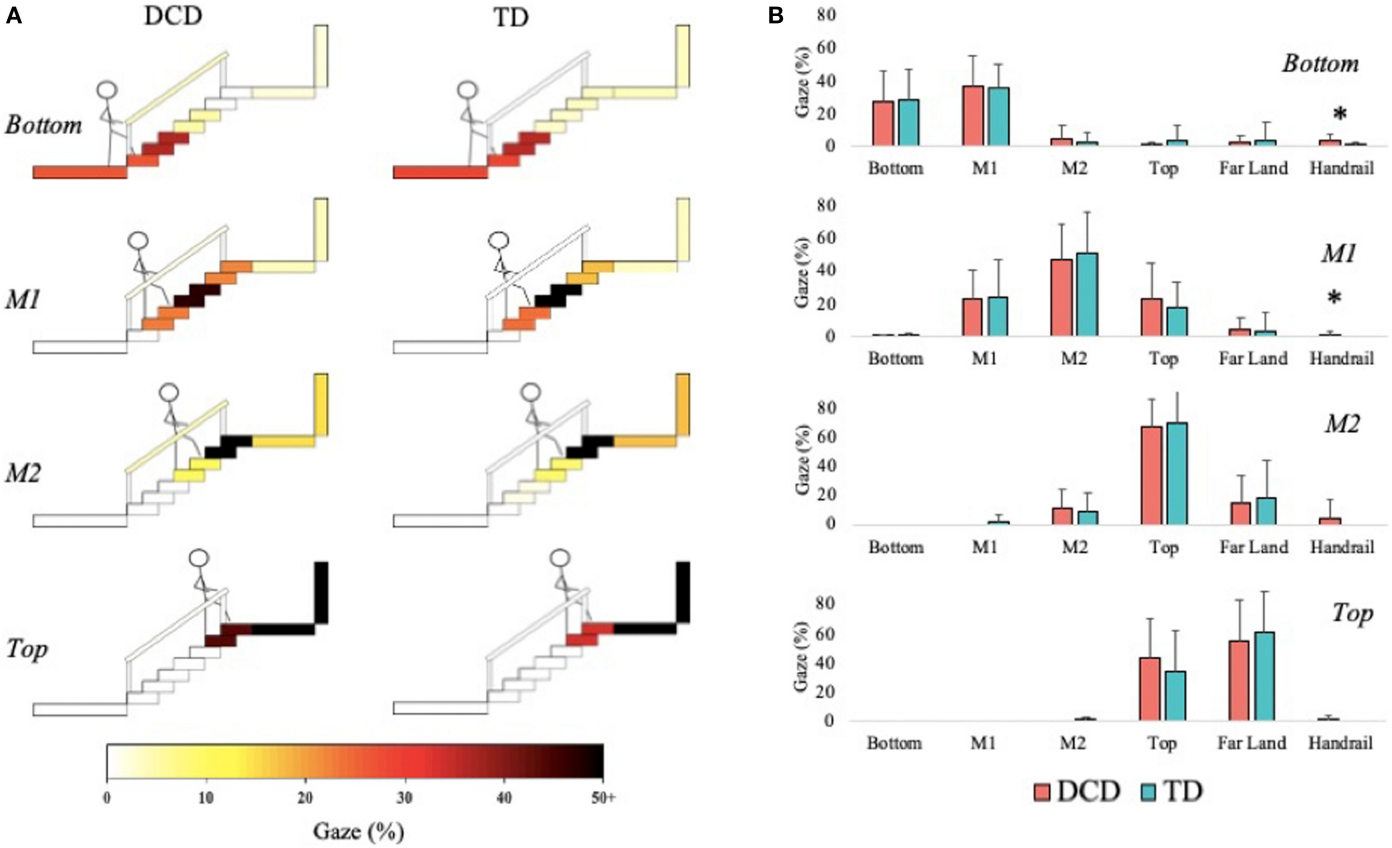
Distribution of gaze fixations on stair features relative to the phase of action for both DCD TD children. **(A)** Adapted from Miyasike-daSilva et al. (2011), each set of stairs shows the participants stepping location (stick figure) and the respective percentage of gaze fixations directed from that location to each of the AOI's. Darker shaded AOI's represent the most fixated regions. **(B)** The mean (±95% CI) percentage of fixations to each AOI and across each task phase are further presented using bar charts for children with and without DCD. Analyses revealed the DCD children to display a greater percentage of fixations toward the handrails than the TD children during the Bottom and M1 task phases (*p < 0.05).

Children with DCD allocated more gaze time than the TD children to the handrails during the initial Bottom phase, *U* = 85.00, *z* = −2.259, *p* = 0.024, *r* = −0.419, and during the M1 phase, *U* = 96.00, *z* = −2.301, *p* = 0.021, *r* = −0.429; Figure 3).

### Stair Descent

#### Anxiety

A Mann-Whitney *U-*test showed that the ranks of state anxiety for the DCD children (Median = 3.00) were significantly greater than the ranks of state anxiety for the TD children (Median = 1.00, *U* = 91, *z* = −2.131, *p* = 0.033, *r* = −0.360).

#### Handrail Use

The children with DCD displayed significantly greater handrail use compared to the TD children, *U* = 91.50, *p* = 0.010, *r* = 0.423. Specifically, three TD children used the handrails, two of whom used the handrail for all 5 trials. In comparison, 15 of the DCD children used the handrails, 11 of whom used the handrails for all 5 trials.

#### Stair Descent Duration

**Figure 4:**
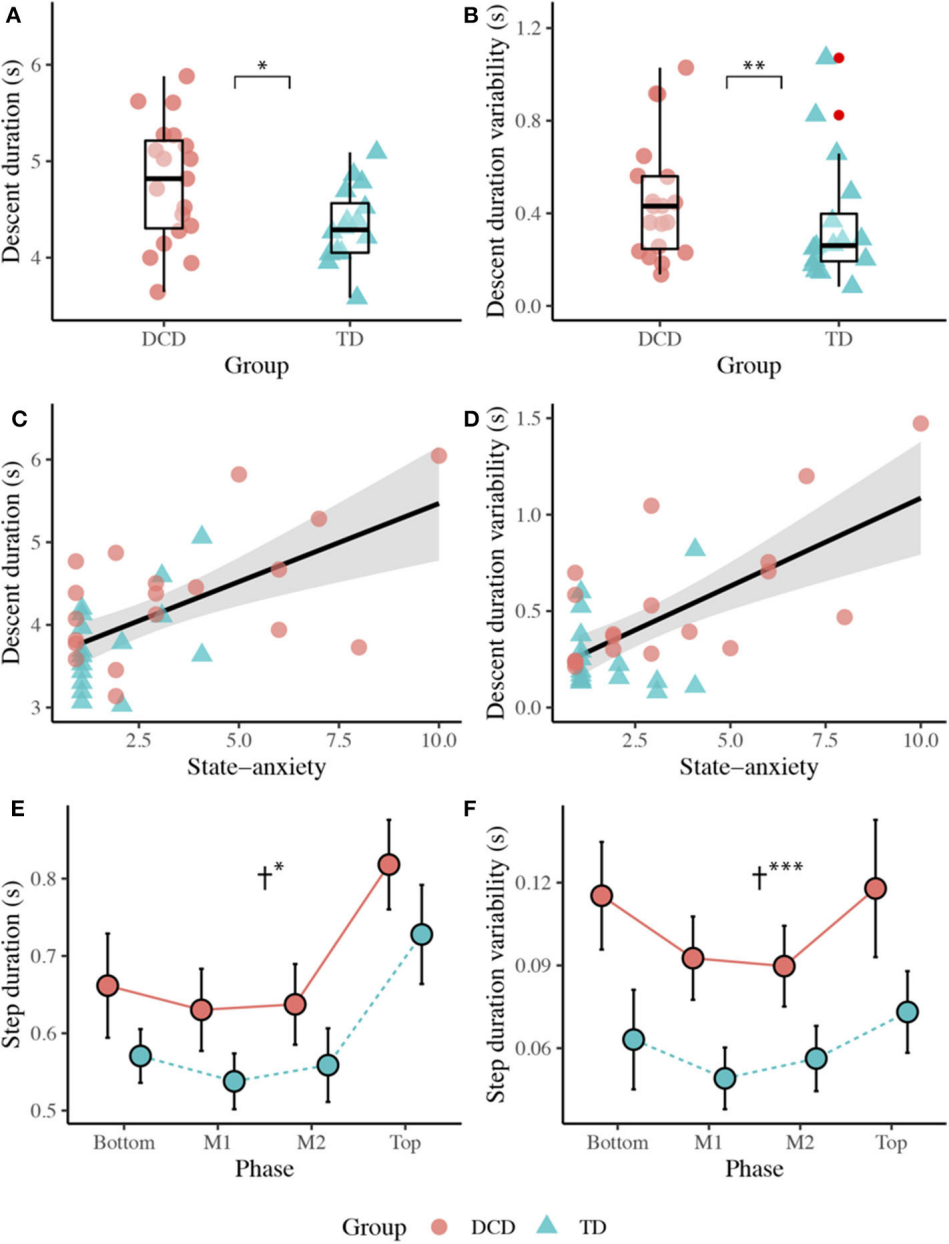
Top panel: Boxplots displaying the median, quartiles, and individual mean time taken to descend the staircase **(A)**, and variability (1 SD across trials) in the time taken to descend the staircase **(B)** for both the DCD and TD groups. Middle panel: Scatter plots displaying the relationship between state anxiety and mean stair descent duration **(C)**, and the relationship between state anxiety and stair descent duration variability **(D)**. Bottom panel: Line plots displaying the mean (±95% CI) time taken to complete each step **(E)** and the variability (1 SD across trials) in time taken **(F)** to complete each step for both the DCD and TD children. † Significant main effect of Group (*p < 0.05, **p < 0.01, ***p < 0.001).

The DCD children (*M* = 4.35 s) took significantly longer than the TD children (*M* = 3.77 s) to descend the staircase, *t*(33) = −2.547, *p* = 0.016, *d* = 0.876. The DCD children (*M* = 0.55 s) also showed greater variability than the TD children (*M* = 0.27 s) in stair descent durations, *U* = 58.00, *z* = −3.113, *p* = 0.002, *r* = −0.526. A Spearman's rank correlation, performed to determine the relationship between state anxiety and stair descent duration, showed state anxiety to display a positive correlation with both stair descent duration (*r*_s_(35) = 0.495, *p* = 0.002) and stair descent duration variability (*r*_s_(35) = 0.409, *p* = 0.015; Figure 4).

#### Step Duration

There was a significant main effect of Group, *F*_(1, 33)_ = 6.343, *p* = 0.017, η_p_^2^ = 0.161, with longer step durations observed in the DCD children (*M* = 687 ms) compared to the TD children (*M* = 599 ms). There was also a significant main effect of Phase, *F*_(2.187, 72.149)_ = 76.038, *p* < 0.001, η_p_^2^ = 0.697, with significantly longest step durations observed during the Top of the staircase (*M* = 773 ms, *ps* < 0.001). There was no Phase x Group interaction, *F*_(3, 99)_ = 0.100, *p* = 0.960, η_p_^2^ = 0.003. A main effect of Group was also observed for step duration variability, *F*_(1, 33)_ = 17.244, *p* < 0.001, η_p_^2^ = 0.343, with greater variability observed in the DCD children (*M* = 104 ms) compared to the TD children (*M* = 60 ms). There was also a significant main effect of Phase, *F*_(3, 99)_ = 9.485, *p* < 0.001, η_p_^2^ = 0.223, best described by a quadratic linear trend (η_p_^2^ = 0.493). Comparisons showed greater step duration variability over the Top (*M* = 9.6 cm) and Bottom (*M* = 8.9 cm) phases of the staircase compared to the M1 (*M* = 7.1 cm) and M2 (*M* = 7.3 cm) phases (*ps* < 0.050). There was no Phase x Group interaction, *F*_(3, 99)_ = 0.952, *p* = 0.419, η_p_^2^ = 0.028 (Figure 4).

### Kinematics

#### Horizontal Heel Clearance

**Figure 5:**
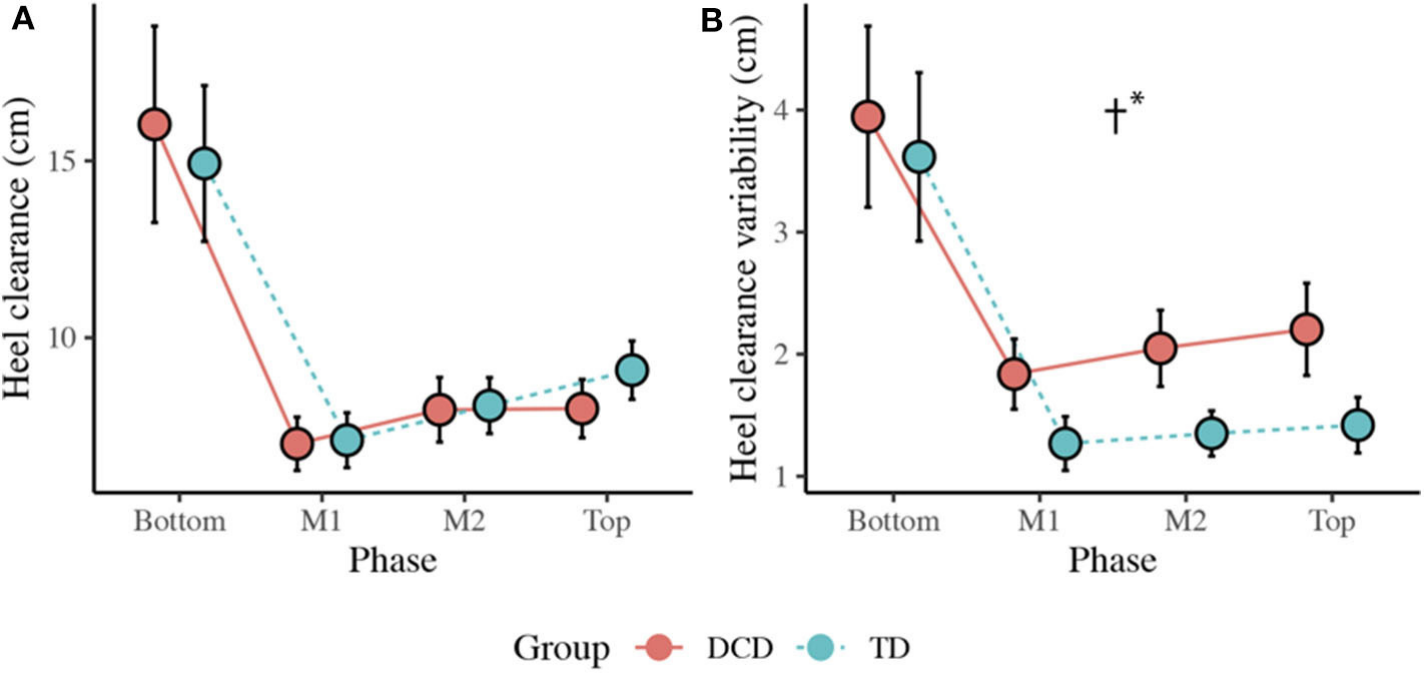
**(A)** Line plots displaying the mean (±95% CI) horizontal heel clearance and mean horizontal heel clearance variability **(B)** for both the DCD and TD children. † Significant main effect of Group (*p < 0.001).

There was no main effect of Group, *F*_(1, 33)_ = 0.005, *p* = 0.942, η_p_^2^ = 0.000, but there was a main effect of Phase, *F*_(3, 99)_ = 66.347, *p* < 0.001, η_p_^2^ = 0.668. Comparisons revealed greatest clearance at the Bottom of the staircase (*M* = 15.5 cm, *ps* < 0.001) and smallest clearance at M1 (*M* = 7.1 cm, *ps* < 0.001) compared to all other phases (Figure 5).

#### Horizontal Heel Clearance Variability

There was a significant main effect of Group, *F*_(1, 33)_ = 13.372, *p* = 0.001, η_p_^2^ = 0.288, with greater variability observed in the DCD children (*M* = 2.5 cm) compared to the TD children (*M* = 1.9 cm). There was also a main effect of Phase, *F*_(1.433, 47.284)_ = 44.423, *p* < 0.001, η_p_^2^ = 0.574, with greatest variability observed at the Bottom of the staircase compared to all other phases (*M* = 3.8 cm, *p* < 0.001; Figure 5).

#### MoS Anteroposterior

There was no main effect of Group, *F*_(1, 32)_ = 0.246, *p* = 0.623, η_p_^2^ = 0.008. There was a main effect of Phase, *F*_(1.827, 58.461)_ = 28.200, *p* < 0.001, η_p_^2^ = 0.468, with lowest MoS values observed at the Top of the staircase (*M* = −8.9 cm) and highest values observed at the Bottom (*M* = −4.0 cm) and M1 (*M* = −4.2 cm) phases.

### Gaze Behavior

#### Fixation Rate/Duration

Independent-samples *t-*tests showed no significant difference between TD (*M* = 2.01) and DCD (*M* = 1.82) groups for mean fixation rate, *t*(27) = 1.530, *p* = 0.138, *d* = 0.571, and no significant difference between TD (*M* = 367.48 ms) and DCD (*M* = 385.15 ms) groups for mean fixation duration, *t*(27) = −0.621, *p* = 0.540, *d* = 0.232.

#### Steps Looked Ahead

**Figure 6:**
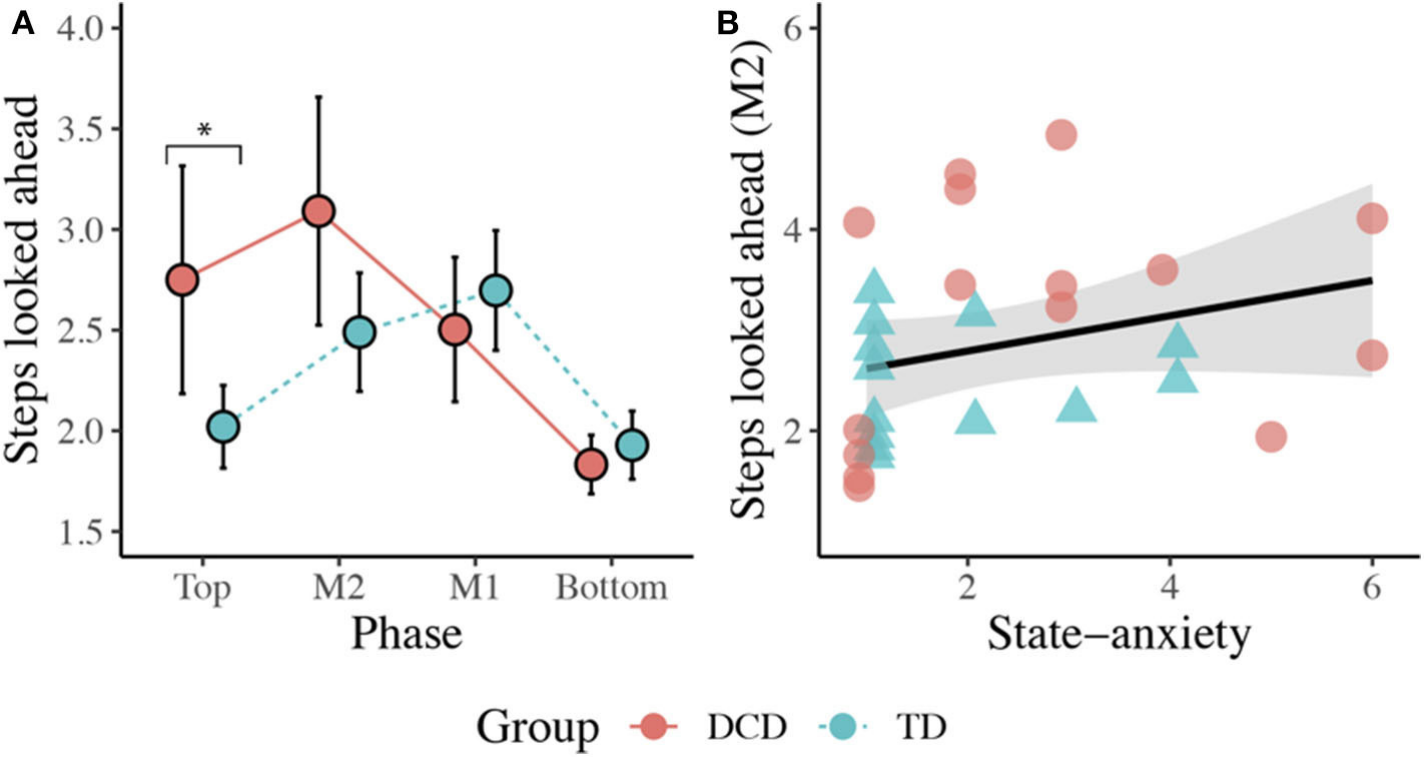
**(A)** Line plot displaying the mean (±95% CI) number of steps looked ahead during each task phase for both the DCD and TD children. *Post-hoc analyses revealed the children with DCD looked significantly more steps ahead than the TD children during the initial Top phase (*p < 0.05). **(B)** Scatter plot displaying the significant positive relationship observed between state anxiety and the mean number of steps looked ahead during the M2 phase (*p* = 0.027).

There was no main effect of Group, *F*_(1, 26)_ = 2.409, *p* = 0.133, η_p_^2^ = 0.085. There was a significant main effect of Phase, *F*_(2.145, 55.770)_ = 16.815, *p* < 0.001, η_p_^2^ = 0.393, with participants looking more steps ahead at the Top (*M* = 2.44) and at M2 (*M* = 2.82) compared to when at the Bottom (*M* = 1.89, *ps* < 0.013) of the staircase. There was also a significant Group x Phase interaction, *F*_(2.145, 55.770)_ = 6.233, *p* = 0.003, η_p_^2^ = 0.193. *Post-hoc* comparisons with Sidak corrections showed that the DCD children (*M* = 2.86) looked significantly more steps ahead than the TD children (*M* = 2.02) during the initial Top phase (*p* = 0.015). A Spearman's rank correlation, performed to determine the relationship between the number of steps looked ahead and state anxiety, showed a positive relationship between state anxiety and the number of steps looked ahead during the M2 phase (*r*_s_(28) = 0.418, *p* = 0.027; Figure 6).

#### Gaze in Action

**Figure 7:**
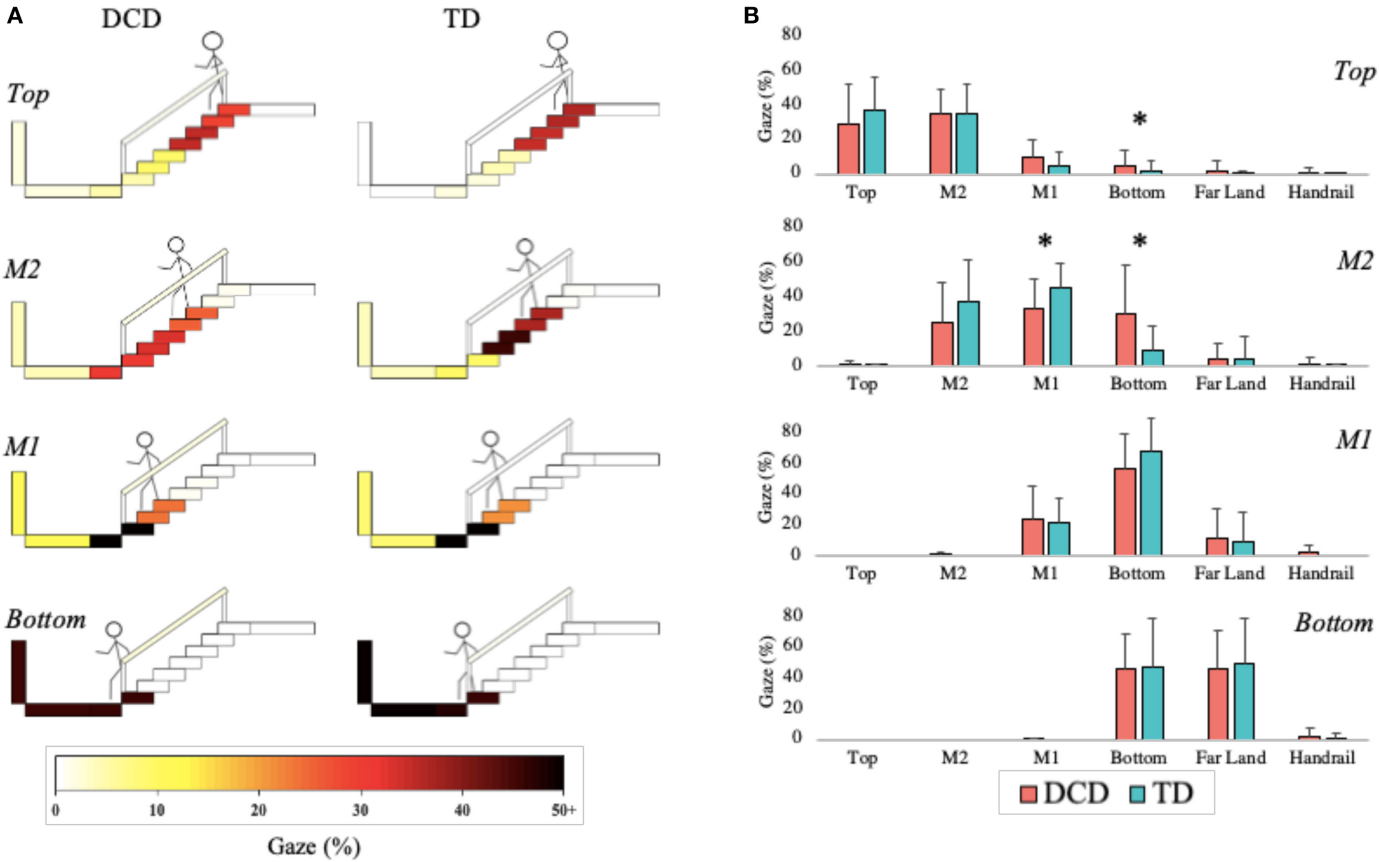
Distribution of gaze fixations on stair features relative to the phase of action for both the DCD and TD children. **(A)** Adapted from Miyasike-daSilva et al. (2011), each set of stairs shows the participants stepping location (stick figure) and the respective percentage of gaze fixations directed from that location to each of the AOI's. Darker shaded AOI's represent the most fixated regions. **(B)** The mean (±95% CI) percentage of fixations to each AOI and across each task phase are further presented using bar charts for both DCD and TD children. Analyses showed that the children with DCD allocated significantly more gaze than the TD children toward the Bottom of the staircase when stood at the Top of the staircase and during the M2 phase (*p < 0.05). In comparison, the TD children allocated significantly more gaze than the DCD children toward M1 during the M2 phase (*p < 0.05).

The DCD children allocated more gaze (*M* = 4.67%) than the TD children (*M* = 1.86%) toward the Bottom of the staircase when they were positioned on the Top phase of the staircase, *U* = 64.50, *z* = −2.086, *p* = 0.037, *r* = −0.387. During the M2 phase, the TD children (45.71%) allocated more gaze than the DCD children (33.33%) to M1, *U* = 59.00, *z* = −2.008, *p* = 0.045, *r* = −0.373, whereas the DCD children (*M* = 30.67%) allocated more gaze than the TD children (*M* = 9.79%) to the Bottom, *U* = 58.00, *z* = −2.074, *p* = 0.038, *r* = −0.385; Figure 7).

## Discussion

The aim of the present study was to provide the first detailed account of the visuomotor control strategies that underpin stair negotiation in children with DCD and account for the possible influence of state anxiety. From our parental confidence survey, we provide the first quantifiable evidence that the parents of children with DCD report significantly lower confidence in their child's ability to maintain balance when walking up and down the stairs—with confidence lowest in their child's ability to navigate the stairs without using the handrails. We also provide evidence that children with DCD experience significantly more stair-related falls than children without DCD. These findings reinforce stair negotiation as a significant issue for children with DCD and reinforce the importance of the present investigation.

### Stair Ascent

When ascending the stairs, the children with DCD walked more slowly (e.g., ~500 ms longer to ascend stairs), displayed greater variability in their walk and step durations, displayed greater frequency of handrail use, and displayed greater toe clearance variability. State anxiety did not differ between groups, nor did their gaze behavior, with both groups maintaining gaze between two and three steps ahead of stepping location, aligning with previous work in older adults (Zietz and Hollands, 2009; Miyasike-daSilva et al., 2011).

More variable walking patterns have been observed in children with DCD previously (Rosengren et al., 2009; Wilmut et al., 2016; Parr et al., 2020) and is commonly taken as a sign of impaired motor control reflecting intrinsic neuromotor noise (Moe-Nilssen and Helbostad, 2005; Smits-Engelsman and Wilson, 2013). However, previous studies have found no difference in walking speeds between DCD and TD children during over ground gait (Wilmut et al., 2016), obstacle crossing (Deconinck et al., 2010) and an adaptive locomotion task (Parr et al., 2020). The slower walking speed adopted by the DCD group, combined with the greater reliance on handrail use, may therefore reflect a protective adaptation to minimize destabilizing momentum (Menz et al., 2004) and explain how the children with DCD were able to maintain similar margins of stability as their TD peers. Indeed, children with DCD have been shown to display difficulties with balance control during walking (Deconinck et al., 2006) and when crossing obstacles (i.e., when no handrails are available; Deconinck et al., 2010). Adopting this more cautious approach may therefore act as an important compensatory strategy that not only promotes stability, but also alleviates concerns related to the fear of falling. However, this adaptive behavior could also reflect the constraints children with DCD experience in lower limb strength and power (Raynor, 2001; Yam and Fong, 2018), given the increased mechanical demands placed on the lower extremities during stair ascent compared to level ground walking (Aldridge et al., 2012).

Despite similar margins of stability, children with DCD still displayed significantly greater toe clearance variability, which may contribute to stair-related falls by increasing the likelihood of accidentally contacting step edges (Hamel et al., 2005). Given the similarity in gaze behaviors between groups, inaccurate stepping in children with DCD during stair ascent is likely to occur regardless of appropriate looking behavior. This finding is consistent with our recent work in over ground precision stepping (Parr et al., 2020) and further implicates an inherent deficit to neuromuscular control of the lower limbs during locomotion in children with DCD (Rosengren et al., 2009). It is also possible that children with DCD are less effective at using the acquired visual information to guide safe and consistent stepping actions (Parr et al., 2020). By adopting a similar “look-ahead” gaze strategy observed in the TD children, the children with DCD are placing similar demands on feedforward and predictive control mechanisms they have previously shown to struggle with (Adams et al., 2014). Maintaining gaze several steps ahead during stair ascent could therefore be detrimental to stepping performance in children with DCD and may increase the risk of stair-related falls.

### Descent

When descending the stairs, the children with DCD again walked more slowly (e.g., ~600 ms longer to descend the stairs), displayed greater variability in their walk and step durations, displayed greater frequency of handrail use, and displayed greater heel clearance variability. However, unlike stair ascent, the children with DCD reported significantly higher levels of state anxiety and utilized a different gaze strategy than the TD children, looking significantly more steps ahead during the initial Top phase of the staircase.

Descending the staircase poses a greater challenge to postural dynamic stability and a greater threat to injury in the event of a fall, compared to stair ascent (Mian et al., 2007). Problems with balance control may therefore increase the fear of falling in children with DCD during stair descent despite compensatory behaviors (e.g., walking slower and using handrails) to maintain stability. As heightened anxiety was not observed during stair ascent, it is possible that motor difficulties in children with DCD may only increase state anxiety when it interferes with the ability to (safely) meet the demands of the task. It is also possible that increased state anxiety may be driven by ruminative thoughts and worries (Ellmers and Young, 2019) given the increased frequency of stair-related falls reported by the parents of children with DCD in the present study. Either way, our results suggest that state anxiety may have influenced the way children walked down the stairs. For example, state anxiety was positively correlated with stair descent duration and variability, suggesting that more anxious individuals walked slower and at more variable speeds when descending the stairs. This is in line with previous research in older adults and may reflect a “stiffening” strategy that is used to avoid potentially destabilizing motor patterns that might inflate the risk of a fall (Young and Mark Williams, 2015). Slower descent speeds have also been observed under conditions that increase the difficulty of visually identifying stair features, such as poor lighting (Thomas et al., 2020) and when faced with ambiguous stair surface patterns (Thomas et al., under review). Slower walking speeds may, therefore, serve to both improve stability and counteract anxiety-related decreases in attentional processing efficiency, allowing more time to extract and process acquired information to guide safe stepping.

State anxiety was also associated with group differences in the spatial allocation of gaze. The children with DCD looked significantly more steps ahead than the TD children during the initial entry (Top) phase which, when considering gaze in action, was seemingly underpinned by a greater tendency to fixate the bottom of the staircase. During the upper-mid-step (M2) phase, the DCD children again spent longer fixating the bottom of the staircase and the number of steps looked ahead was positively correlated with state anxiety. This tentatively suggests an anxiety-specific response that may bias gaze toward the planning of future stepping actions over the accurate execution of ongoing stepping commands (Chapman and Hollands, 2006) and may reflect a hypervigilance toward distant aspects of the environment that are perceived to pose a threat to balance (Young and Mark Williams, 2015). Maintaining gaze further along the travel path may, therefore, better serve balance in children with DCD by simplifying the extraction of pertinent information from optic flow and providing peripheral vision of the lower limbs and stairs (Zietz and Hollands, 2009). However, looking further ahead is likely to place an increased reliance on an internalized representation of stair dimensions and the use of predictive motor control. Given substantial evidence that children with DCD have difficulties generating and implementing predictive models of action (c.f. Adams et al., 2014), it is possible that this anxiety-driven gaze response may be contributing to increased heel clearance variability and the risk of falls.

### Practical Implications to Improve Stair Safety

Taken together, our results highlight significant differences in the visuomotor control strategies that underpin stair negotiation in children with and without DCD. However, it is unclear at this point whether the visuomotor control strategies observed in the DCD group are actually mitigating or contributing to their increased frequency of stair-related falls. Future attempts to answer this question could, therefore, have practical implications for the optimisation of stair safety in children with DCD. For example, eye-movement training has been used to improve the coordination and performance of throwing and catching in children with DCD (Słowiński et al., 2019) and increase stepping accuracy in older adults when navigating obstacles (Young and Hollands, 2010). Manipulating eye-movement behavior during stair negotiation could therefore help determine an “optimal” gaze strategy that could subsequently be trained to aid stair negotiation. Similarly, movement training interventions have been used to improve functional strength and balance in children with DCD (Ferguson et al., 2013; Jelsma et al., 2014; Bonney et al., 2017) and to improve balance and reduce the fear of falling in older adults (Li et al., 2005; Schmid et al., 2010). Understanding how improved functional strength and balance influence visuomotor control and anxiety would shed light on the mechanisms that underpin stair problems in children with DCD. Furthermore, evidence suggests that focusing attention internally, toward the conscious online processing of motor commands, can result in slower, less efficient and more unstable locomotion (Mak et al., 2020; Young et al., 2020). Future research could therefore consider the interplay between overt (spatial allocation of gaze) and covert attentional processes to provide a more holistic understanding of the attentional strategies that may differentiate DCD and TD stair navigation. Finally, it would be interesting to determine whether difficulties and/or anxieties with stair negotiation in children with DCD are related to the reduced confidence of their parents. It is possible that some parents may overcome concerns relating to injury by preventing each child from being exposed to potentially destabilizing situations (i.e., stairs without handrails), thus hindering the development of these task-specific skills.

## Limitations

The results of this study should be considered with respect to several limitations that may stimulate the questions to be addressed in future work. For example, our sample includes a relatively wide age range (8–15 years) that is likely to encompass children of varying developmental maturation. As the development of visually guided stepping goes through distinct changes throughout these developmental years (Mowbray et al., 2019) we invite caution when extrapolating our findings to children of all ages. We also acknowledge the limitations of self-reported state anxiety in children given developmental aspects of emotional self-perception (Smith et al., 2006). However, the positive relationship we observed between anxiety and stair descent duration is consistent with previous literature and reinforces the utility of these simple inventories. Yet, future work would still benefit from attempts to objectively capture a physiological state anxiety response to compliment measures of self-report and overcome the limitations of ordinal data. Similarly, our binary measure of handrail use fails to quantify the precise handrail onset, duration, laterality, and contact force, each of which are required to determine the full extent of handrail dependency in children with DCD. Furthermore, it is important to recognize that handrails are not always available to aid stability. Future work should, therefore, explore how the manipulation of handrail use influences the risk/fear of falling in children with DCD during stair negotiation. It is also interesting to acknowledge that our task lacks the environmental complexity children are likely to face when navigating the stairs in the real world. For example, navigating a busy staircase at school will likely require the foveation of other people's walking behavior to avoid collision (Jovancevic-Misic et al., 2007). Similarly, using a mobile phone will draw attention away from the stairs and place greater demands on peripheral vision (Ioannidou et al., 2017). Understanding how a concurrent task affects stair safety could therefore have significant implications for clinicians managing children with DCD. Finally, whilst margin of stability provides a comprehensive assessment of dynamic balance, this measure has not been routinely used in the DCD literature. Future work may therefore benefit from complimenting margin of stability with more familiar measures of balance control (for review, see Verbecque et al., in press) to triangulate issues with stability.

## Conclusion

In conclusion, the results of this study show that (a) safe stair negotiation is a significant and anxiety-inducing task that children with DCD struggle with, and (b) that there are clear differences in the visuomotor control strategies that underpin stair negotiation in children with and without DCD. Overall, it appears that children with DCD overcome difficulties with balance control, and successfully maintain stability, by walking slower and relying heavily on handrail use. However, children with DCD still display evidence of significantly greater step-edge clearance variability than TD children, which possibly increases the risk of a fall. Unlike stair ascent, children with DCD report heightened anxiety prior to stair descent and look further along the staircase during the initial entry phase. However, it is unclear at this point whether these anxiety related alterations to gaze are detrimental to stair negotiation safety and contribute to the frequency of falls.

## Data Availability Statement

The raw data supporting the conclusions of this article will be made available by the authors, without undue reservation.

## Ethics Statement

The studies involving human participants were reviewed and approved by Liverpool John Moores Ethics Committee. Written informed consent to participate in this study was provided by the participants' legal guardian/next of kin. Written informed consent was obtained from the minor(s)' legal guardian/next of kin for the publication of any potentially identifiable images or data included in this article.

## Author Contributions

MH, RF, and GW acquired the funding. MH, RF, GW, and NT edited the manuscript. MH, RF, GW, and JP designed the study. JP and NT undertook the data collection. JP completed the statistical analysis, figure preparation, and the first draft of the manuscript. All authors approved the final version of the manuscript for submission.

## Conflict of Interest

The authors declare that the research was conducted in the absence of any commercial or financial relationships that could be construed as a potential conflict of interest.
